# Morphological and Molecular Characterization of *Ottolenchus isfahanicus* n. sp. (Tylenchomorpha: Tylenchoidea) from Rhizosphere of Gramineous Plants in Isfahan Province, Iran

**DOI:** 10.2478/jofnem-2023-0011

**Published:** 2023-04-21

**Authors:** Zeinab Mahboubi, Mojtaba Keramat, Ebrahim Pourjam, Mohammad Reza Atighi, Ilenia Clavero-Camacho, Joaquín Abolafia, Pablo Castillo, Majid Pedram

**Affiliations:** Department of Plant Pathology, Faculty of Agriculture, Tarbiat Modares University, Tehran, Iran; Instituto de Agricultura Sostenible (IAS), Consejo Superior de Investigaciones Científicas (CSIC), Avenida Menéndez Pidal s/n, 14004, Córdoba, Spain; Departamento de Biología Animal, Biología Vegetal y Ecología, Universidad de Jaén, Campus Las Lagunillas, s/n, 23071, Jaén, Spain

**Keywords:** new species, *Ottolenchus facultativus*, phylogeny, SSU rDNA, taxonomy

## Abstract

A new species from the family Tylenchidae was recovered in the grasslands of Isfahan province, Iran, and is herein described based on morphological and molecular data. *Ottolenchus isfahanicus* n. sp. is mainly characterized by having a faintly annulated cuticle; elongated, slightly sigmoid amphidial apertures developed metacorpus with distinct valve under LM; vulva located at 69.472.3% of body length; large spermatheca about 2.75 times of corresponding body width; and elongated conoid tail with broadly rounded tip. SEM observations showed that the lip region is smooth; the amphidial apertures are elongated, slightly sigmoid slits; and the lateral field is a simple band. It is further characterized by 477-to-515-μm-long females with delicate 5.7-to-6.9-μm-long stylets with small, slightly posteriorly sloping knobs, as well as functional males, in the population. The new species closely resembles *O. facultativus*, but is separated from it based on morphological and molecular data. It was further morphologically compared with *O. discrepans*, *O. fungivorus*, and *O. sinipersici*. The phylogenetic relationships of the new species with other relevant genera and species were reconstructed using near-full-length sequences of small subunit and D2-D3 expansion segments of large subunit (SSU and LSU D2-D3). In the inferred SSU phylogeny, the newly generated sequence of *Ottolenchus isfahanicus* n. sp. formed a clade with two sequences of *O. sinipersici* and sequences assigned to *O. facultativus* and *O. fungivorus*. In the inferred LSU phylogeny, the three newly generated sequences of the new species and LSU sequences of *O. sinipersici* and *O. discrepans* formed a clade.

The family Tylenchidae Örley, 1880 represents an abundant and diverse group of nematodes. Ecologically, they are one of the main parts of soil fauna, and may constitute up to 30% of the nematodes in a given soil sample (Ferris and Bongers, 2006; Yeates and Bird, 1994; Qing and Bert, 2017). The subfamily Tylenchinae Örley, 1880 currently includes 12 genera (Siddiqi, 2000; Qing and Bert, 2017). The taxonomy of the genus *Ottolenchus* Husain & Khan, 1967 has been revised several times. Geraert and Raski (1987), in their revision of the genus *Filenchus* Andrássy, 1954, noted that the number of lateral lines is an unreliable generic character, and synonymized it with *Filenchus.* However, Siddiqi and Lal (1992) emphasized the characters differentiating *Ottolenchus* from *Filenchus*, and urged that *Ottolenchus* was a reliable taxon by virtue of the longitudinal, curved and sigmoid amphidial apertures, originating at the lateral lip areas and extending over most of the lip region, and narrow lateral field with two incisures. Siddiqi (2000), Decraemer and Hunt (2006, 2013), Qing and Bert (2017), and Hosseinvand et al. (2021) also considered *Ottolenchus* to be a valid genus.

Poshtkooh forests are located in the vicinity of Fereydunshahr city, Isfahan province, and are covered with diverse flora, e.g., *Quercus* L., *Crataegus* L. etc. During our study, a population of *Ottolenchus* representing a new species was recovered from the grasslands of Fereydunshahr, and we described and illustrated it based upon morphological and molecular data.

## Materials and Methods

*Soil sampling, nematode extraction and morphological characterization:* 180 soil samples were collected from the grasslands of Isfahan province, Iran, between 2019-2022. The samples were placed in plastic bags, transferred to the nematology laboratory of Tarbiat Modares University, and maintained at cool temperature conditions. Nematodes were extracted from soil samples using the tray method (Whitehead and Hemming, 1965); handpicked under a Nikon SMZ1000 (Nikon, Tokyo, Japan) dissecting microscope; heat-killed by adding boiling, 4% formaldehyde solution; and transferred to anhydrous glycerin according to de Grisse (1969). Drawings and morphological studies were performed using a drawing tube attached to a Nikon E600 light microscope, and digital drawings were completed using CorelDraw software version 2020. The light microphotographs of the fresh individuals and mounted specimens were prepared using an Olympus BX51 microscope equipped with a digital DP72 camera (Olympus) and differential interference contrast (DIC) optics. Siddiqi (2000) and original descriptions of *Ottolenchus* spp. were used as the taxonomic framework and morphological comparisons resources, respectively.

*Scanning electron microscopy (SEM):* Few specimens preserved in glycerine were selected for SEM observation according to Abolafia (2015). The nematodes were hydrated in distilled water, dehydrated in a graded ethanol-acetone series, critical point dried, coated with gold, and observed with a Zeiss Merlin microscope (5 kV) (Zeiss, Oberkochen, Germany).

*DNA extraction, polymerase chain reaction (PCR) and sequencing:* Seven specimens were examined on temporary slides to confirm their identity, and then individual DNA samples from each specimen were extracted in 15 μl of TE buffer (10mM Tris-Cl, 0.5mM EDTA; pH 9.0). DNA samples were stored at -20 °C until they could be used as PCR templates. The SSU rDNA was amplified using two pairs of primers which yielded overlapping fragments: forward 1096F (5´-GGTAATTCTGGAGCTAATAC-3´) and reverse 1912R (5´-TTTACGGTCAGAACTAGGG-3´) primers to amplify the first fragment, and forward 1813F (5´-CTGCGTGAGAGGTGAAAT-3´) and reverse 2646R (5´-GCTACCTTGTTACGACTTTT-3´) primers to amplify the second fragment (Holterman et al., 2006). Primers for the LSU D2-D3 amplification were the forward primer D2A (5´-ACAAGTACCGTGAGGGAAAGTTG-3´) and the reverse primer D3B (5´-TCGGAAGGAACCAGCTA CTA-3´) (Nunn, 1992). The PCR mixture (35 ml) contained the following components: 17 μl *Taq* DNA Polymerase 2× Master Mix RED, 2-mM MgCl2 (Ampliqon, Odenese, Denmark), 10 μl distilled water, 1.5 μl of each primer, and 5 μl of DNA template. The thermocycling program was as follows: denaturation at 95°C for 4 min, followed by 35 cycles of denaturation at 94°C for 30 sec, annealing at 52°C for 40 sec, and extension at 72°C for 80 sec. A final extension was performed at 72°C for 10min. The PCR products were sequenced with a DNA multicapillary sequencer (Model 3130XL Genetic Analyzer; Applied Biosystems, Foster City, CA, USA), using the BigDye Terminator Sequencing Kit v.3.1 (Applied Bio-systems) at the Stab Vida sequencing facility (Caparica, Portugal) with the same primers used for amplification, and deposited into the GenBank database under the accession number OP972577 for SSU, and the accession numbers OQ025275, OQ025276 and OQ025277 for LSU rDNA D2-D3.

*Phylogenetic analyses:* The chromatogram files of the newly generated sequences were checked using Chromas Lite 2.1.1 (https://technelysium.com.au/wp/chromas/), edited, trimmed, and assembled manually. The SSU sequence of *Ottolenchus isfahanicus* n. sp. was compared with other available sequences in the GenBank database using the basic local alignment search tool (BLAST). Relevant sequences (mostly belonging to the subfamily Tylenchinae) used in SSU phylogeny in previous studies (Monemi et al., 2022) were selected for inferring the SSU tree. Two sequences of Aphelenchoidea Fuchs, 1937, namely *Bursaphelenchus mucronatus* Mamiya & Enda, 1979 (AY284648) and *Aphelenchoides fragariae* Ritzema Bos, 1890 (AY284645), were selected as outgroups. The LSU sequences of *Ottolenchus isfahanicus* n. sp. were compared with other available sequences in the GenBank database using BLAST homology search program. The maximal number of relevant sequences as already used in our previous studies (Monemi et al., 2022; Mortazavi et al., 2021) were included in LSU phylogeny.

The SSU and LSU datasets were aligned using the Q-INS-i algorithm of the online version of MAFFT (version 0.91b) (https://mafft.cbrc.jp/alignment/server) and the resulting alignments were edited manually using MEGA6 (Tamura et al., 2013). The best-fit model of nucleotide substitution was selected using PAUP*/MrModeltest.2 (Nylander, 2004). The Akaike-supported model, a general time-reversible (GTR) model including among-site rate heterogeneity and estimates of invariant sites (GTR + gamma [G] + invariant [I]), was selected and used in both phylogenies. Bayesian analyses were performed using MrBayes 3.1.2 (Ronquist and Huelsenbeck, 2003) by running the chains for 5 × 10^6^ generations. After discarding burn-in samples, the remaining samples were retained for further analyses. The Markov chain Monte Carlo (MCMC) method within a Bayesian framework was used to estimate the posterior probabilities of the phylogenetic trees (Larget and Simon, 1999) using the 50% majority rule. The convergence of model parameters and topology was assessed based on an average standard deviation of split frequencies and potential scales reduction factor values. Adequacy of the posterior sample size was evaluated using autocorrelation statistics as implemented in tracer v.1.6 (Rambaut and Drummond, 2009). The output files of the trees were visualized using Dendroscope v3.2.8 (Huson and Scornavacca, 2012) and digitally drawn in CorelDRAW software version 2020.

## Results

### Systematics: Ottolenchus isfahanicus n. sp.

(Figs. 1-3; Table 1).

**Figure 1:**
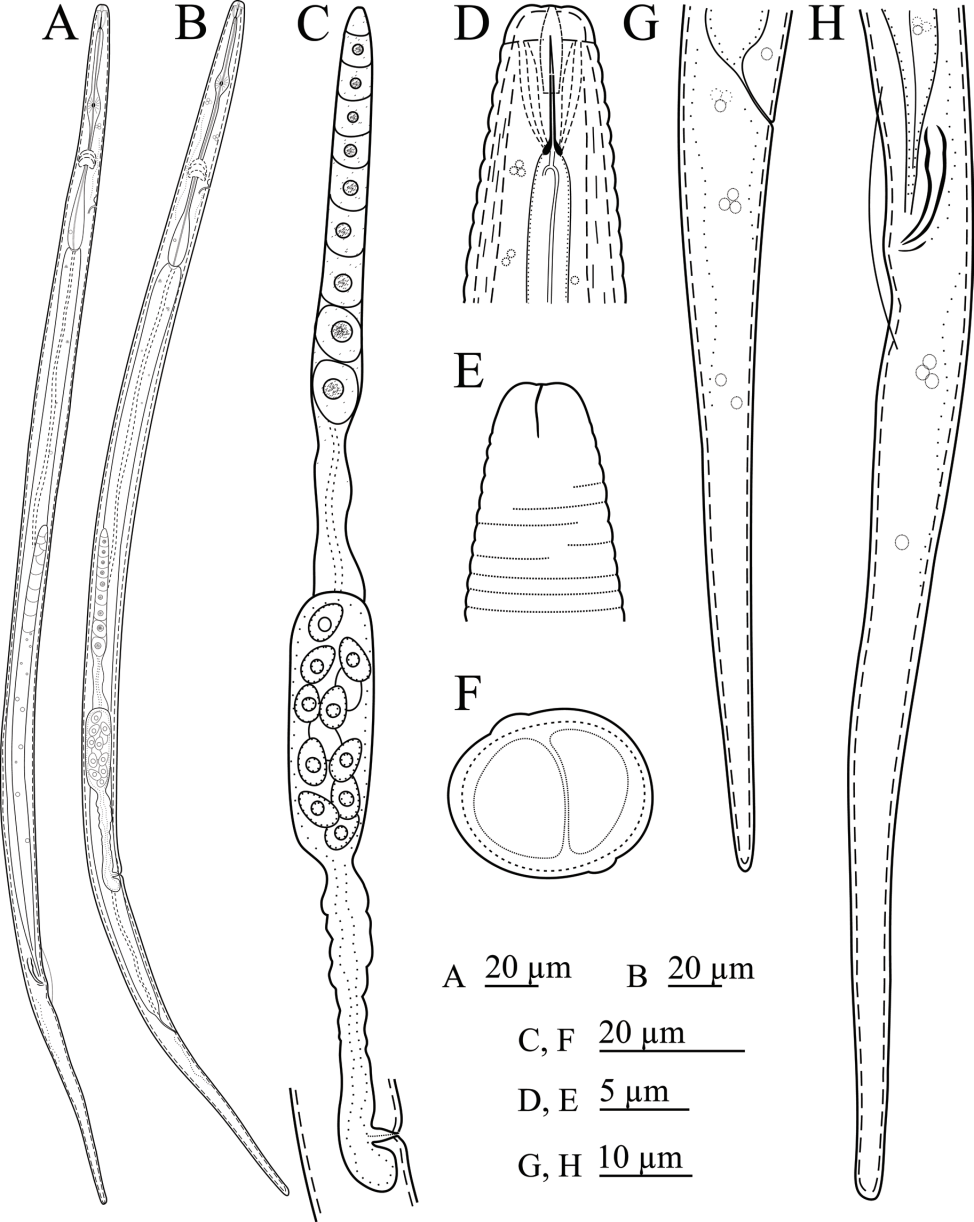
Line drawings of *Ottolenchus isfahanicus* n. sp. (A, H: Male; B–G: Female). (A, B) Entire body. (C) Reproductive system (D) Anterior body region (E) Amphidial aperture. (F) Cross section at mid-body. (G, H) Tail and posterior body region.

**Figure 3:**
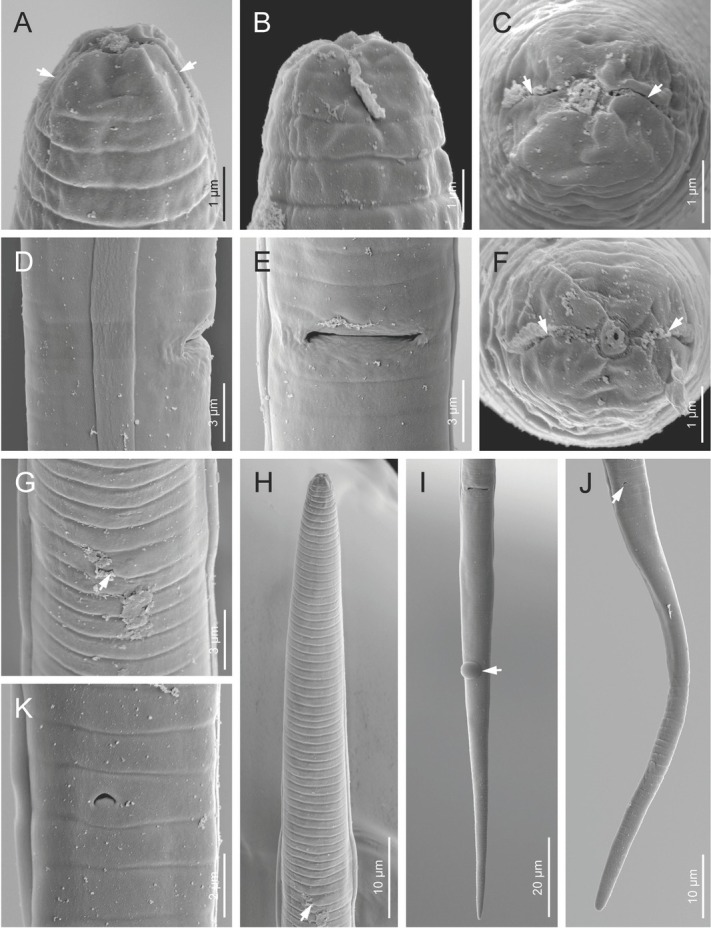
Scanning electron microphotographs of *Ottolenchus isfahanicus* n. sp. (Female). (A–C, F) Lip region in ventral, lateral and face views, respectively (arrows pointing to the amphidial apertures). (D, E) Vulva in lateral and ventral views, respectively. (G) Excretory pore (arrow). (H) Anterior body region in ventral view (arrow pointing to the excretory pore). (I) Posterior body region (arrow pointing to the anus with liquid feces). (J) Tail (arrow pointing to the anus).

**Table 1:** Morphometrics of *Ottolenchus isfahanicus* n. sp. All measurements are in μm and in the form mean ± SD (range).

**Characteristic**	**Holotype**	**Paratypes**	
	**Female**	**Females**	**Males**
N		13	3
L	505	493.2 ± 12.1 (477-515)	430.3 ± 12.4 (416-438)
L’	420	409.6 ± 10.3 (392-428)	353.3 ± 12.4 (339-361)
a	36.0	36.0 ± 2.2 (32.5-39.6)	37.8 ± 1.8 (35.7-39.1)
b	6.0	5.4 ± 0.2 (5.2-5.7)	4.9 ± 0.2 (4.7-5.0)
c	6.0	5.9 ± 0.4 (5.3-6.7)	5.6 ± 0.2 (5.4-5.6)
c’	9.0	8.8 ± 0.7 (7.5-9.7)	9.7 ± 0.7 (8.9-10.2)
V or T	71.0	70.8 ± 1.0 (69.4-72.3)	80.0 ± 0.0 (81-82)
V’	85.0	85.2 ± 1.0 (83.3-86.6)	-
Lip region height	2	1.6 ± 0.2 (1.3-1.9)	1.7 ± 0.1 (1.5-1.7)
Lip region width	5	4.4 ± 0.3 (4.0-4.9)	4.5 ± 0.1 (4.4-4.6)
Stylet	6	6.3 ± 0.4 (5.7-6.9)	6.1 ± 0.3 (5.8-6.4)
Conus	2	1.8 ± 0.2 (1.5-2.2)	2.1 ± 0.2 (1.9-2.3)
Excretory pore	77	75.6 ± 2.2 (72-79)	68.3 ± 2.1 (66-70)
MB	38	37.5 ± 2.4 (32-40)	37.3 ± 2.5 (35-40)
Pharynx	91	91.3 ± 2.3 (88-95)	88.0 ± 5.3 (82-92)
Anterior end-vulva	358	394.2 ± 8.3 (337-361)	353.3 ± 12.4 (339-361)
Body width (BW)	14	13.7 ± 0.8 (13-15)	11.4 ± 0.2 (11.1-11.6)
Anal body width	10	9.5 ± 0.5 (9-10)	8 ± 0.6 (7.52-8.6)
Vulva-anus	62	60.5 ± 4.8 (55-68)	77 ± 0 (77-77)
Tail length	85	83.5 ± 6.0 (72-92)	11.1 ± 0.6 (10.5-11.5)
Tail/V-A	1	1.4 ± 0.2 (1.1-1.7)	-
PUS	15	14.3 ± 1.2 (12.2-16.4)	-
Spicules	-	-	11.1 ± 0.6 (10.5-11.5)
Gubernaculum	-	-	2.2 ± 0.9 (1.2-2.9)
Bursa length	-	-	20.3 ± 1.0 (19.3-21.2)

### Description

*Female:* Body is slightly ventrally curved after fixation. Cuticle has faint annuli, sometimes indistinct under the light microscope (Fig. 2A,D,E). Lateral fields are simple bands formed by two faint incisures under SEM. Lip region is smooth under SEM, continuous with body. Amphidial apertures are slightly sigmoid longitudinal slits (Fig. 3B,C,F), barely visible under LM. Oral opening is surrounded by six labial papillae located in a hexagrammate plate. Stylet is slender, delicate; conus is one-third of total stylet, with knobs sloping posteriorly. The pharyngeal dorsal gland orifice (DGO) is close to the knobs. Pharyngeal procorpus is slender, metacorpus is well developed with distinct valve. Isthmus is narrow and slender, pharyngeal bulb pyriform, cardia small and conical. Nerve ring encircles isthmus at about the middle. Excretory pore with distinct duct is at level with anterior pharyngeal bulb. Hemizonid is scarcely distinct, located at nerve ring level. Intestine is simple; anus and rectum are functional. Reproductive system is monodelphic-prodelphic; ovary is outstretched with oocytes arranged in a single row; oviduct is tubular; spermatheca is offset, large, and elongate-ellipsoid, longer than twice the corresponding body width, with large spheroid sperm cells; the border of crustaformeria and uterus is indistinct; vagina is perpendicular to body axis; vulva is a small slit (Fig. 3E); and postvulval uterine sac (PUS) is about as long as the vulval body diameter. Tail is conoid and elongate, with a broadly rounded tip.

**Figure 2:**
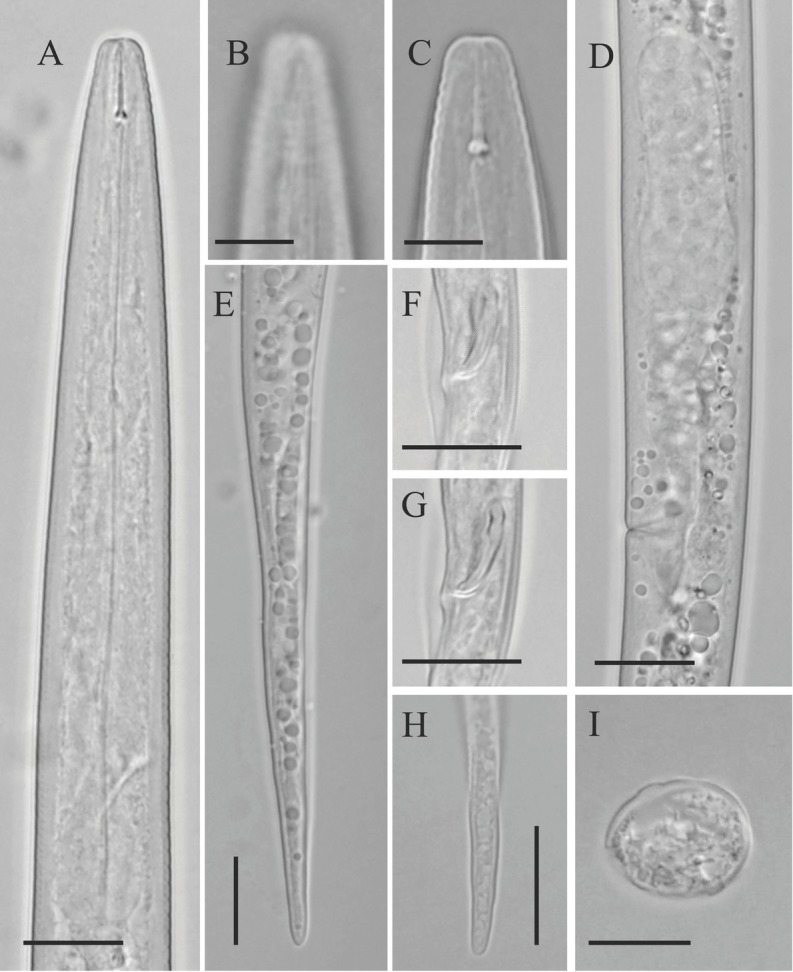
Light microphotographs of *Ottolenchus isfahanicus* n. sp. (A–E, I: Female; F, G, H: Male). (A–C) Anterior body region showing pharynx, amphidial aperture and stylet, respectively. (D) Spermatheca. (E, H) Tail and tail tip (F, G) Spicule and bursa. (I) Lateral field at midbody. (All scale bars: B, C = 5 μm, rest = 10 μm).

*Male:* Common and functional (sperm observed inside female spermatheca). Similar to females in general morphology, except in sexual characters. Spicules are tylenchoid, slender, slightly arcuate. Gubernaculum is simple and small. Tail is similar to that of females. Bursa is cloacal and small.

*Type habitat and locality:* The new species was recovered from a soil sample collected from the rhizosphere of gramineous plants in the grasslands of Fereydunshahr, Isfahan province, Iran, on 22 May 2021. The global position system (GPS) coordinates are 33°30.0440’N and 50°22.5960’E.

*Type material:* Holotype female and eleven paratype females and three males (in six slides with the accession codes WT3928-WT3933) were deposited into the WaNeCo nematode collection (http://www.waneco.eu/), The Netherlands. One paratype female was deposited in the nematode collection of the Departamento de Biología Animal, Biología Vegetal y Ecología, University of Jaén (slide code IRN012-01). The LSID code of this publication is: urn:lsid:zoobank.org:pub:07D142D7-57EE-4315-801C-DFE77AA849A9.

*Etymology:* The specific epithet refers to the Isfahan province, where the new species was found.

### Diagnosis and relationships

The new species is mainly characterized by having a faintly annulated cuticle, appearing smooth under LM; two lines in lateral field which form a plain band; smooth lip region; amphidial apertures longitudinal, slightly sigmoid slits; stylet delicate with posteriorly sloping knobs; spermatheca large, elongate-ellipsoid, about 2.75 times body width at corresponding region; and males present, functional. The new species was morphologically compared with the type population of relevant species of the genus. It could be separated:

From *O. discrepans* Andrássy, 1954 by having a longer body (477-515 *vs* 393-407 μm), greater a value (32.5-39.6 *vs* 27.2-31.6), greater c value (5.3-6.7 *vs* 4.0-4.4), greater V value (69.4-72.3 *vs* 61.2-64.1), and elongate-conoid tail with broadly rounded tip *vs* filiform.

From *O. facultativus* Szczygieł, 1969 by having a faintly annulated cuticle (*vs* coarsely annulated), larger spermatheca (further than twice corresponding body width *vs* about as wide as corresponding body width), greater V value (69.4-72.3 *vs* 65.9-67.6), and greater c value (5.3-6.7 *vs* 4.6-5.0).

From *O. fungivorus* Bert, Okada, Tavernier, Borgonie & Houthoofd, 2010 by having a longer body (477-515 *vs* 284-331 μm), greater a value (32.5-39.6 *vs* 21.9-30.9), smaller c´ value (7.5-9.7 *vs* 8.2-12.5), greater V value (69.4-72.3 *vs* 58.1-66.6), shorter PUS (12.2-16.4 *vs* 5.1-5.5 μm), and a large spermatheca *vs* undifferentiated.

From *O. sinipersici* Hosseinvand, Eskandari, Abolafia, Karegar, Ghaderi, Majd Taheri & Hajializadeh, 2021 by having a shorter (72-92 *vs* 113-135 μm long), elongate conoid straight tail with broadly rounded tip (*vs* filiform, hook-like or coiled at the end), shorter stylet (5.7-6.9 *vs* 10.3-11 μm), shorter pharynx (88-95 *vs* 89-113 μm), and greater V value (69.4-72.3 *vs* 66.9-69.5).

*Molecular phylogenetic status:* Sequencing of the SSU and LSU rDNA D2-D3 fragments of the new species yielded a single 1,460-nt-long SSU (OP972577), and three 670-nt-long LSU (OQ025275, OQ025276, OQ025277) sequences. The BLAST search using the newly generated SSU sequence revealed that it has 90.79% identity with FJ949564 assigned to *Filenchus* sp. and 90.54% identity with MZ044896 (*Ottolenchus sinipersici*). The identity value with other SSU sequences was less than 90.54%.

The BLAST search using the newly generated LSU sequences revealed that their identity with all currently available LSU sequences is less than 85%. The highest identity belonged to *Sigmolenchus sinuosus* (MK611960, MK611959, MK611958). In the SSU phylogenetic tree (Fig. 4), the original sequence of the new species, *Ottolenchus isfahanicus* n. sp. and two sequences of *O*. *sinipersici* (MZ044896, MZ044897) formed a clade along with the sequences assigned to *O. facultativus* (KJ869310) and *O. fungivorus* (FJ949564). This clade is in sister relation with the clade including several sequences of *Malenchus* spp. and *Miculenchus* spp. Sequences of *Ottolenchus discrepans* (AB473565, KX156305) and *O. longiurus* Siddiqi & Lal, 1992 (KJ869337) form basal lineages to the abovementioned group.

The LSU phylogenetic tree (Fig. 5) grouped the original sequences of the new species, *Ottolenchus sinipersici* n. sp. (MN822618, MZ044898) and sequences assigned to *O. discrepans* (KX156321, KX156315, KX156317) in a single clade, which is in sister relation with a clade including several sequences of *Malenchus* spp.

**Figure 4:**
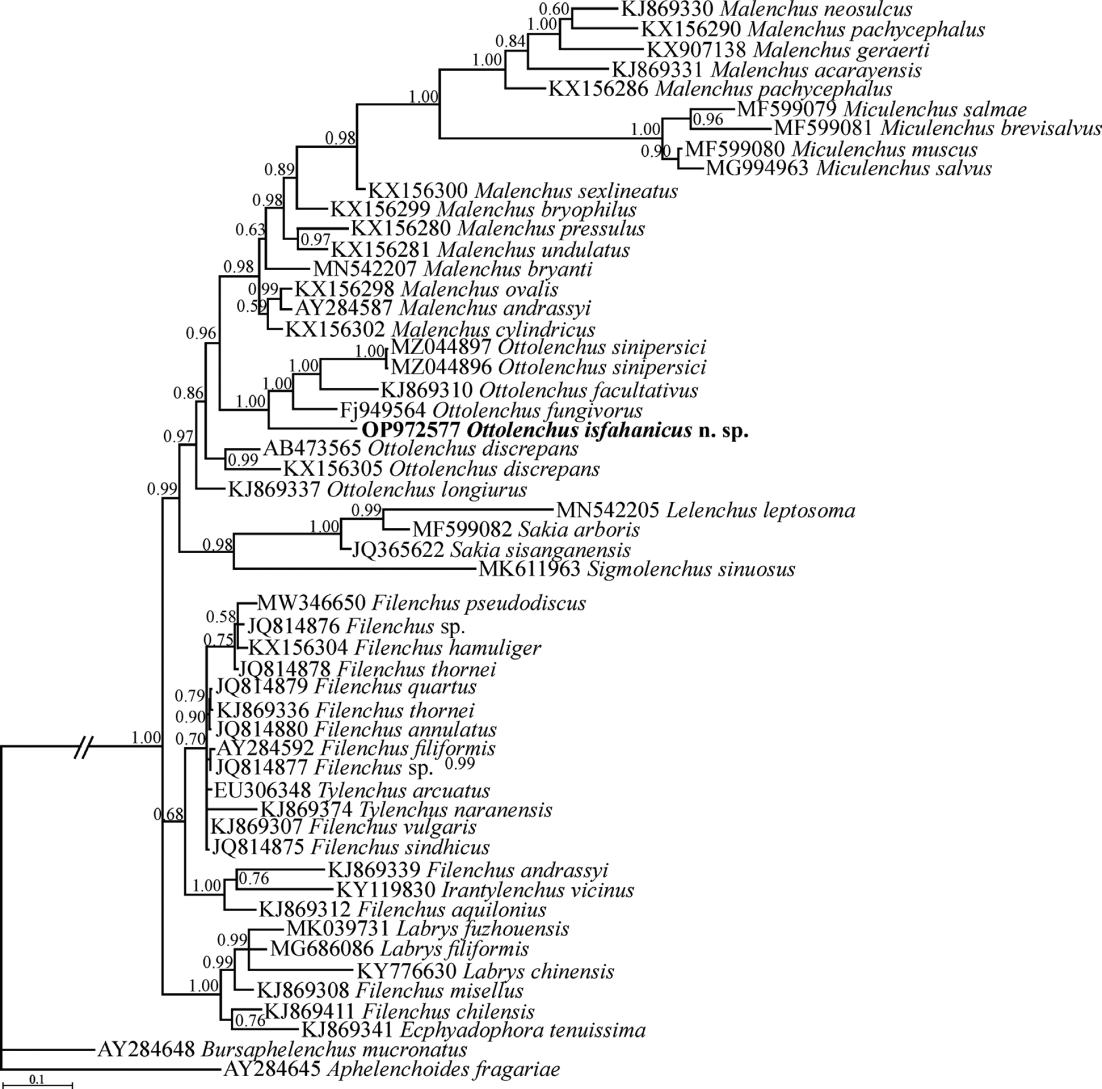
Bayesian 50% majority rule consensus tree inferred from the SSU rDNA of *Ottolenchus isfahanicus* n. sp. under the GTR + G + I model (-ln L = 22872.6152; freq A = 0.1925; freqC = 0.2759; freqG = 0.3011; freq T = 0.2305; R(a) = 1.1309; R(b) = 2.9105; R(c) = 1.1816; R(d) = 0.9105; R(e) = 4.1153; R(f) = 1.0000; Pinva = 0.2877; Shape = 0.4556). Bayesian posterior probability values are given for corresponding clades. The new species is in bold font. GTR, general time-reversible; G, gamma; I, invariant; rDNA, ribosomal DNA; SSU, small subunit.

## Discussion

The genus *Ottolenchus* is now a valid and well-established genus (Qing and Bert, 2017; Hosseinvand et al., 2021). It is paraphyletic in SSU, and monophyletic in LSU phylogenies, after recent studies based on available sequences of its representatives (Hosseinvand et al., 2021). On the other hand, currently only few representatives of *Ottolenchus* have been sequenced and further information will better depict its phylogenetic nature in the future.

In the present study, the new species was compared with the original description of the morphologically closest species. The morphometrics of most species of Tylenchidae have been expanded following numerous reports (Geraert, 2008); however, most of the original and subsequent reports lack molecular sequences, making the identification of most of the species questionable. Monemi et al. (2022) stated that most of the sequences assigned to *Boleodorus thylactus* Thorne, 1941 probably belong to cryptic forms, and therefore, topotype sequences of the species are needed to decide on their identity. Likewise, topotype sequences have been used to prove the status of sequences assigned to *Xiphinema brevicolle* Lordello and Da Costa, 1961 (Lazarova et al., 2019) and *X. robbinsi* Pedram, Niknam & Decraemer, 2008 (Jahanshahi Afshar, 2019). For all of the above, the molecular data of the type series, e.g., topotypes, are needed for correct identification of given species in future. In the current study, the new species was separated from *Ottolenchus fungivorus*, the tentative closest species, based on the morphometric data, and their independent lineages were corroborated by their position on the SSU tree.

**Figure:**
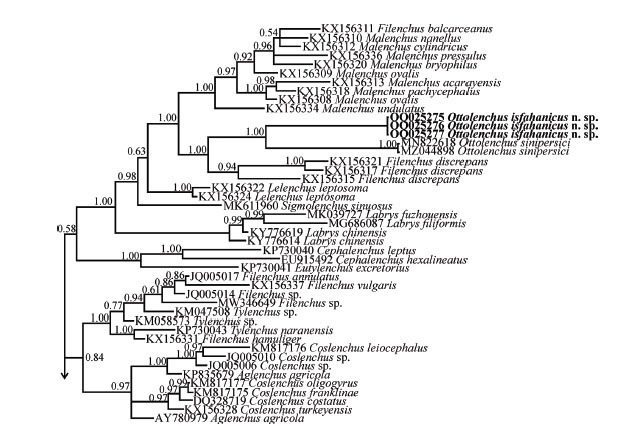


**Figure 5:**
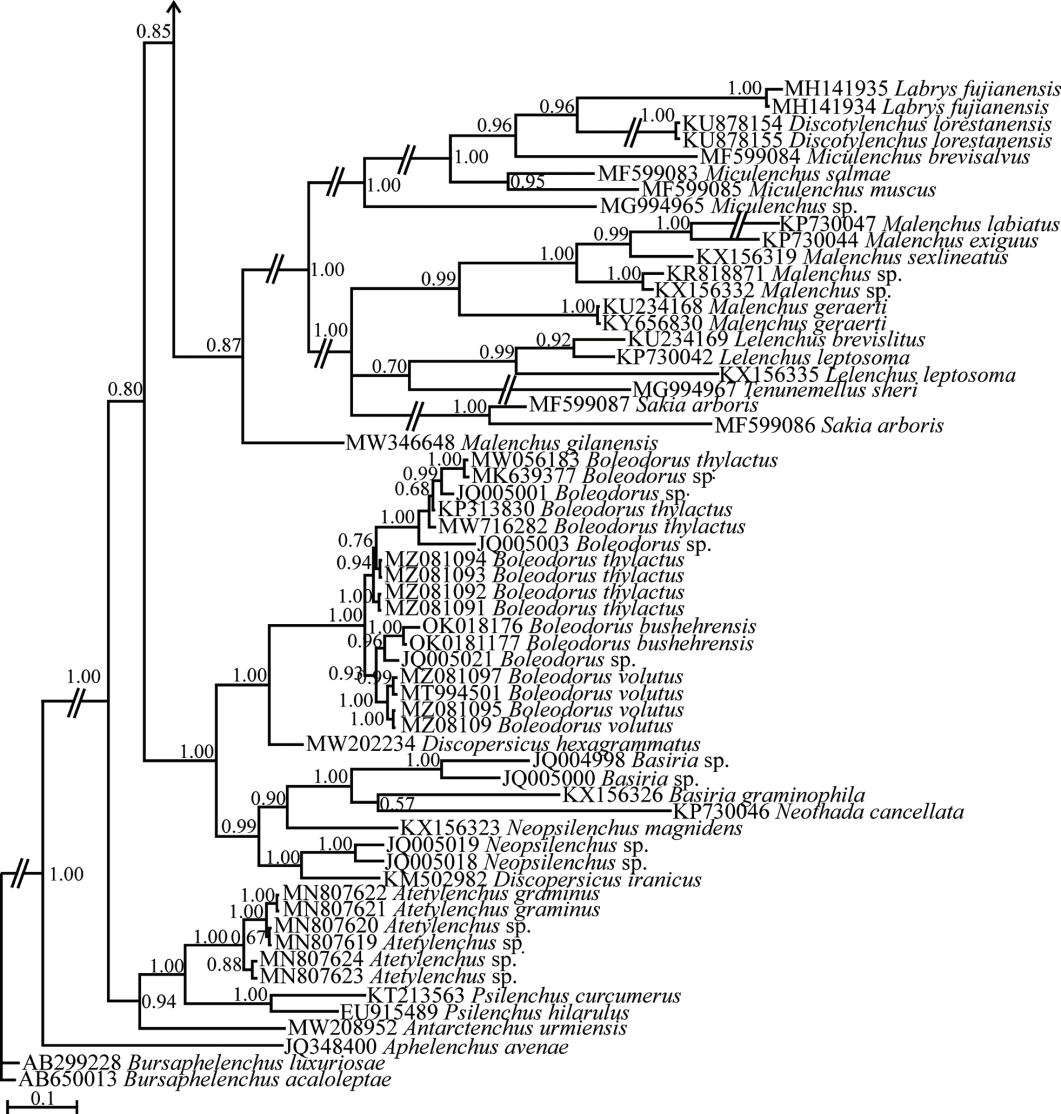
Bayesian 50% majority rule consensus tree inferred from the LSU rDNA D2–D3 sequences of *Ottolenchus isfahanicus* n. sp. under the GTR + G + I model (-ln L = 24658.7734; freq A = 0.1395; freqC = 0.2523; freqG = 0.3373; freq T = 0.2709; R(a) = 1.1364; R(b) = 3.0722; R(c) = 1.3293; R(d) = 0.7313; R(e) = 3.9786; R(f) = 1.0000; Pinva = 0.1104; Shape = 0.6954). Bayesian posterior probability values are given for the corresponding clades. The new species is in bold font. GTR, general time-reversible; G, gamma; I, invariant; LSU, large subunit; rDNA, ribosomal DNA.
